# Water-soluble fullerene derivatives mitigate cranial radiation-induced neuroinflammation and cognitive dysfunction

**DOI:** 10.1007/s10544-026-00818-w

**Published:** 2026-05-11

**Authors:** Naren Gundapaneni, Iona Hill, Lydia W. T. Cheung, Khadijeh Koushki, Alyssa Fausnaught, Phuoc Minh Quan Mai, Arjun Vasan, Prapannajeet Biswal, Ashutosh Tripathi, Anilkumar Pillai, Vijayasree V. Giridharan, Tatiana Barichello, Yuri Mackeyev, Sunil Krishnan

**Affiliations:** 1 Vivian L. Smith Department of Neurosurgery, University of Texas Health Science Center, Houston, TX 77030 USA; 2 Faillace Department of Psychiatry and Behavioral Sciences, University of Texas Health Science Center, Houston, TX 77030 USA

**Keywords:** Fullerene, C_60_, C_60_-ser, Radiation, Neuroprotection, Cognitive impairment, Inflammation

## Abstract

Radiotherapy is widely used to treat malignant and benign brain tumors. A major constraint, however, is the low tolerance of normal brain tissue to radiation. Cranial irradiation-induced injury manifests as inflammatory responses and neurodegeneration, which jointly contribute to progressive cognitive decline. These complications can restrict the delivery of optimal therapeutic doses, cause persistent neurological symptoms, and ultimately compromise quality of life. Thus, neuroprotective agents have emerged as a strategy to safeguard normal tissues from radiation-induced neurotoxicity. This study investigated the efficacy of a highly soluble [60]fullerene (C_60_) derivative, C_60_-ser, a carbon-based nanomaterial, to mitigate radiation-induced brain injury. Oral administration of C_60_-ser 24 h after cranial irradiation of male C57BL/6 mice restored spatial working memory, as evidenced by significantly improved performance in the Y-maze test conducted 45 days after irradiation. At the cellular level, immunohistochemical analysis revealed that irradiation alone activated microglia for up to 84 days after exposure, indicative of generating a sustained pro-inflammatory microenvironment. Critically, this activation was markedly alleviated in mice treated with C_60_-ser; CD68 reactivity was restored to levels similar to unirradiated mice. Together, these behavioral and molecular findings suggest that C_60_-ser can mitigate radiation-induced neurotoxicity by suppressing neuroinflammatory responses when administered 24 h after cranial irradiation.

**Graphical abstract:**

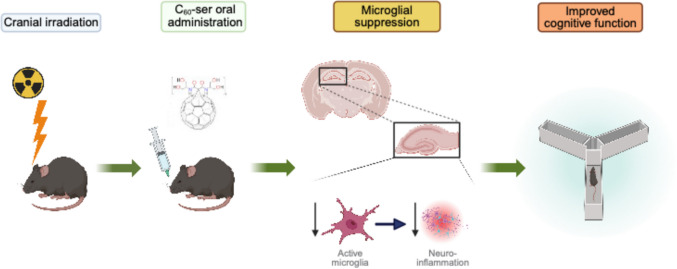

**Supplementary Information:**

The online version contains supplementary material available at 10.1007/s10544-026-00818-w.

## Introduction

Ionizing radiation (IR) plays an integral role in the treatment of primary and metastatic brain tumors in adult and pediatric patients. However, IR-related neurocognitive impairment is a major clinical problem in neuro-oncology (Turnquist et al. 2020). Irradiation of deep-seated intracranial tumors inevitably exposes surrounding healthy brain tissue to radiation. This risk is further exacerbated by radioresistance of many brain tumors, which often necessitate dose escalation or re-irradiation (Helis et al. 2022; Kelley et al. 2016). Consequently, cumulative radiation exposure increases the likelihood of normal tissue toxicity and neurocognitive decline. IR-induced cognitive decline has been shown to occur in up to 50—90% of patients who survive > 6 months after treatment with high-dose radiotherapy for primary or metastatic brain tumors (Cramer et al. 2019; Makale et al. 2017). Clinical manifestations range from learning and memory dysfunction to deficits in executive function to severe dementia, significantly affecting the quality of life of long-term survivors. Patients with head and neck cancer may also receive substantial incidental radiation exposure to brain tissue and experience cognitive decline (Welsh et al. 2014). Although advanced modalities including proton beam therapy, stereotactic radiosurgery, and intensity-modulated radiation therapy have significantly improved conformal avoidance of normal brain tissue while retaining the spatial precision of radiation delivery (Rahman et al. 2022), neurocognitive impairment may still occur, for example, in verbal memory, motor dexterity, and processing speed (Chow et al. 2024; Kahalley et al. 2020). These persistent effects underscore the need for mechanistic insights and the development of neuroprotective strategies (Ryan et al. 2011).

Targeting the molecular mechanisms underlying IR-induced cognitive toxicity represents a promising neuroprotective strategy. Specifically, neuroinflammation plays a central role in the development of IR-induced brain injury (Turnquist et al. 2020; Wilke et al. 2018). Radiation exposure initiates a neuroinflammatory response primarily through oxidative damage caused by the generation of free radicals and reactive oxygen species, which lead to activation of inflammatory signaling pathways and microglia, the brain-resident macrophages (Lumniczky et al. 2017; Son et al. 2015). Persistently activated microglia produce inflammatory cytokines, which perpetuate inflammation and suppress neurogenesis, particularly within the hippocampus (Leskinen et al. 2025; Lull & Block 2010; Makale et al. 2017; Monje et al. 2002). The inflammatory cascade is further amplified by reactive astrocytes and infiltrating peripheral immune cells (Lee et al. 2023; Liddelow & Barres 2017; Rezai-Zadeh et al. 2009), linking radiation-induced oxidative stress to chronic neuroinflammation and the subsequent cognitive impairment. Given these mechanisms, reducing the neurotoxic microenvironment by targeting inflammation and oxidative stress may mitigate cognitive dysfunction (Monje et al. 2003; Wilke et al. 2018).

Free radical-scavenging nanotherapeutics have been explored as potential neuroprotective agents (Talaat et al. 2025; Y. Zhang et al. 2025). Among these, [60]fullerene (C_60_), which is a polyhedral caged carbon allotrope, exhibits exceptional radical-scavenging ability owing to its pseudo-aromatic structure due to delocalization of π-electrons over its carbon core and high electron affinity (Castro et al. 2017). However, their application has been limited by inherent hydrophobicity and poor aqueous dispersibility (Castro et al. 2017; Sun et al. 2026). While previous in vivo studies have examined the radioprotective and antioxidant effects of C_60_ in whole-body radiation mouse models with readouts on survival, oxidative stress, and DNA damage (Brown et al. 2010; Cai et al. 2010; Gudkov et al. 2019), there is a lack of research directly assessing its effects on cognitive performance and neuroinflammation following cranial radiation exposure.

In this study, we report the neuroprotective effects of C_60_-ser administration in a mouse model of whole-brain irradiation. As a novel, highly soluble and orally bioavailable C_60_ derivative, C_60_-ser exhibits excellent blood–brain barrier permeability and localizes to the brain for more than a week (Raoof et al. 2012). The favorable pharmacokinetic profile, broad therapeutic window, and evidence of in vivo efficacy reported herein provide strong support for the potential of C_60_-ser in neuroprotection against radiation-induced brain damage.

## Materials and methods

### Synthesis of serinol malonamide tetraacetate and C_60_-ser

*Preparation of serinol malonate*: 182 g of serinol (2-amino-1,3-propanediol, TCI America, Portland, OR) was added to 132 g of dimethyl malonate (Alfa Aesar, Ward Hill, MA) dissolved in 2 L of isopropyl alcohol (VWR, Radnor, PA). The mixture was stirred at 65 °C for 96 h and then cooled to room temperature. White precipitate of serinol malonate (*N*,*N*'-bis-(1,3-dihydroxypropan-2-yl)-malonamide) was washed with isopropyl alcohol, air-dried, and purified by recrystallization from boiling methanol (VWR Chemicals BDH, Radnor, PA). Yield: 240 g (96%). Purity was controlled by measuring the melting point (134 °C).

Acetylation of serinol malonate was then achieved by dropwise addition of pyridine (60 g, Alfa Aesar) to a solution of 25 g of serinol malonate in 100 g of acetic anhydride (Alfa Aesar) under stirring, while maintaining the temperature below 40 °C through control of pyridine addition rate. After evaporation of all volatile products under vacuum, the remaining solid residue was dissolved in 250 mL of acetonitrile (VWR Chemicals BDH), filtered with Whatman glass microfiber filters (Grade GF/F, Sigma-Aldrich, St. Louis, MO), and cooled in a dry ice box overnight. White precipitate was separated by using the filter funnel with fritted disc (coarse porosity, Synthware) under vacuum and dried with a flow of moisture-free nitrogen. Second recrystallization was performed by dissolving in 100 mL of methyl-*tert*-butyl ether (Fisher Chemical, Pittsburgh, PA) and methylene chloride (VWR Chemicals BDH) at 4:1 ratio at 40 °C, prior to cooling to room temperature. Yield: 23 g (55%). Purity of the serinol malonamide tetraacetate (*N*,*N*’-bis(1,3-diacetyloxypropan-2—yl)propanediamide) was controlled by thin-layer chromatography and melting point measurement (96 °C).

*Preparation of C*_*60*_*-ser*: 2.16 g of C_60_ (3 mM, ATS-MER LLC, Tucson, AZ) and 10 g of carbon tetrabromide (30 mM, Acros Organics, Geel, Belgium) were dissolved in 1.5 L of anhydrous toluene (VWR Chemicals BDH) under nitrogen. With stirring, 200 mL of anhydrous chloroform (VWR Chemicals BDH) was added. Solutions of 11.3 g (27 mM) of serinol malonamide tetraacetate in 50 mL of chloroform and 4 g (27 mM) of DBU (1,8-diazabicyclo[5.4.0]undec-7-ene, Sigma-Aldrich) in 5 mL of toluene were added from two separate reservoirs. After 24 h, the reaction mixture was purified by silica gel absorption using “RediSep R_f_ Gold” normal phase silica flash columns (particle size 20 to 40 µm, average pore size 60 Å)(Teledyne ISCO, Lincoln, NE), with subsequent liquid chromatography performed in parallel on five columns using a gradient with chloroform (A) and methanol (B), from pure A to A:B = 19:1 mixture. The fraction containing C_60_-ser acetate, hexakis-adduct, was identified by comparing to the reference hexakis-adduct on RediSep TLC plates (Teledyne ISCO), dried, and subjected to hydrolysis. Combined yield: 1.5 g (16%).

Next, C_60_-ser acetate (1.5 g, 0.46 mM) dissolved in 50 mL of dioxane was mixed with 1 mL of 37% hydrochloric acid, with stirring at 40 °C for 35 min. After volatile compounds were removed in vacuum, the solid C_60_-ser residue was dissolved in 10 mL of distilled water, and neutralized with 0.5 M sodium bicarbonate solution to pH 6.5. The final C_60_-ser product (hexakis-adduct) was isolated by multiple dialyses in deionized water, with scaling-up achieved using a Spectra/Por Tube-A-Lyzer dialysis system. Oligomeric impurities were isolated through the ultra-pure cellulose ester 3.5 kDa dialysis membrane, whereas salts and other low molecular weight impurities were removed with 1 kDa dialysis membrane. The resulting C_60_-ser solution was dried under vacuum, and preserved as solid at 4 °C. Combined yield: 0.3 g (30%).

*Verification of C*_*60*_*-ser purity:* Initial quality verification was performed by using silica gel matrix TLC plates (Sigma-Aldrich) and methanol as mobile phase by comparing with previously synthesized C_60_-ser standard, characterized by Bruker AutoFlex Speed MALDI ToF mass spectrometry. Further quality control was carried out on an Agilent 1200 HPLC (pump G1312B, autosampler G1329A, evaporative light scattering detector ELSD G4260B) equipped with a Jupiter 5 µm C18 300 Å guard column and a Kinetex 2.6 µm C18 100 Å column (100 × 2.1 mm) (Phenomenex, Torrance, CA). All analyses were repeated at least 5 times with consistent peak parameters. Data were acquired and processed using OpenLab C.01.10 software (Agilent).

### Animals and treatments

Four-week-old male black C57BL/6 mice were purchased from The Jackson Laboratory (Bar Harbor, ME), allowed to acclimate for 72 h and cared for in the UT Health Houston Animal Facility with the help of The Center for Laboratory Animal Medicine and Care. Mice were housed, 5 per cage, in a room maintained at constant temperature and humidity under a 12-h light and dark cycle. Mice were fed regular chow diet with access to water ad libitum. All experimental protocols were reviewed and approved (AWC-22—0098) by the Institutional Animal Care and Use Committee (IACUC) at UTHealth. Mice were randomly assigned to one of the six treatment groups (Control, C_60_-ser only, 10 Gy, 10 Gy + C_60_-ser, 30 Gy, 30 Gy + C_60_-ser), with each group having a sample size of 16 and 10 for cohort 1 and 2, respectively (Fig. 2a). Six out of 10 of mice from each treatment group in cohort 2 were also part of cohort 1. Mice were monitored for health and weighed 3 times a week. Weights were normalized to the initial starting weight pre-irradiation.

The 4.5-week-old mice were anesthetized with ketamine (80—120 mg/kg) mixed with xylazine (5—10 mg/kg) and were placed on their side under lead shielding with a circular cut-out to treat a 7—mm area of the brain behind the eye while sparing oronasopharyngeal mucosa, similar to the geometry described previously (Eley et al. 2020). This area was defined previously on computed tomographic imaging of representative mouse brains and the lead cut-out was placed directly on the head to expose an area of the brain that spared unnecessary treatment of the eyes or the oronasopharynx. A dose of 10 Gy or 30 Gy was administrated by a RS 2000 small animal biological irradiator (Rad Source Technologies, Buford, GA) with dose quantification being done through built-in calculations on the RS 2000. Mice in the groups receiving 0 Gy (Control and C_60_-ser only) were taken to the radiator room and anesthetised but no irradiation was administered. These mice were maintained under anesthesia for the same length of time as the irradiation treated mice. All mice were then given atipamezole (1 mg/kg) as a reversal agent and placed on a warming pad to recover. Mice were monitored post-anesthesia until full recovery. Twenty-four hours post-irradiation, mice were given either C_60_-ser (700 mg/kg) or 100 µl PBS as control via oral gavage. All mice were monitored after oral gavage for any adverse effects.

### Object in place (OIP) and novel object recognition (NOR)

Behavior tests were performed at 45 ± 5 days post-C_60_-ser treatment (cohort 1) and lasted for 5 days. On each test day, mice were allowed to acclimate to the testing room for 30 min. Test days 1—4 consisted of a day for acclimatization to testing chamber, training, OIP, and then NOR. At the 84-day timepoint (cohort 2), OIP was not performed; test days 1—3 consisted of acclimatization to testing chamber, training, and NOR. Six mice from each treatment group which underwent testing at the 45-day timepoint were included in the tests at the 84-day timepoint. The two tests were conducted following the standard protocols with an inter-trial interval of 24 h and trial time of 10 min (Denninger et al. 2018). All objects and stages were cleaned with 70% ethanol and allowed to dry before the next trial. All trials were recorded with an overhead camera with the recordings stored directly in a computer. The mice underwent the tests at the same time of day as previous days. The EXPLORE program was used to quantify the time a mouse interacted with an object according to the instructions (Ibanez et al. 2023).

On day 1 (habituation), mice were placed at the lower left corner in an empty 60 cm × 40 cm × 50 cm testing apparatus were allowed to freely explore for 10 min (Giridharan et al. 2022).

On day 2 (familiarization/acquisition), the mice were placed at the same location and testing apparatus as on day 1, alongside 2 identical objects for 10 min as shown in Fig. 2a. Exploration time was determined using EXPLORE and defined as time spent with object 1 + time spent with object 2.

On day 3 (OIP test), mice were place in chamber at the same location as days 1 and 2. Two objects placed in the apparatus were the same objects as in day 2, but the location of one of the objects was moved as shown in Fig. 2a. Mice were allowed to explore for 10 min. The discrimination index was determined using EXPLORE and defined as (time spent with object in new location—time spent with unmoved object)/(time spent with object in new location + time spent with unmoved object). Exploration time was also calculated as time spent with object in new location + time spent with unmoved object.

On day 4 (NOR test/retention), the NOR test was done on day 4 for cohort 1 or day 3 for cohort 2 (Giridharan et al. 2023). The mouse was placed at the same location in chamber as the previous days. The object that had not changed location on the previous day was replaced with a novel object as shown in Fig. 2a. For cohort 2 in which OIP was not conducted, one of the objects was replaced with the novel object, while the locations stayed the same. The other object (familiar/old) was kept the same. Mice were allowed to explore for 10 min. The discrimination index was determined using EXPLORE and defined as (time spent with novel object—time spent with familiar object)/(time spent with novel object + time spent with familiar object). Exploration time was also calculated as time spent with novel object + time spent with familiar object.

### Quantification of behavior in OIP and NOR

Separate EXPLORE models were built for each of the following: training day, OIP, NOR for cohort 1, and NOR for cohort 2. Models were tested on behavioral videos and required refining through multiple iterations using the correction command as outlined by the developer (https://github.com/Wahl-lab/EXPLORE). Final models reported accuracy above 97%, with a minimum of 50,000 + frames labelled for each model. The same model was used for all six groups. The object interaction time was determined for the first 9 min. Results were documented and discrimination index was calculated as described above. Mice with exploration times less than 9 s in OIP or NOR test were excluded from analysis of that testing day (Chou et al. 2016).

### Y-maze

On test day 5 for cohort 1 or day 4 for cohort 2, the mouse was placed in the Y-maze according to the standard Y-maze protocol with a trial time of 10 min (Kraeuter et al. 2019). Testing was performed at the same time of day as previous tests. Mice were brought into the testing room and allowed to acclimatize to the room in their cages for 30 min. Each arm of the Y-maze (32 cm × 6 cm × 20 cm) had distinct visual cues positioned above the maze wall at the end of the arm (Tripathi et al. 2025). After testing each mouse, the maze was cleaned with 70% ethanol and allowed to dry before the next trial.

Y-maze data were analyzed by manual review of the video recordings and mouse entries into an arm were documented. Arm entry was counted when all 4 limbs of a mouse entered an arm. If a mouse went into an arm and then to the middle and back to the same arm, it was considered as one entry rather than two separate, distinct entries. An alternation was counted when the mouse entered the 3 different arms during a trial. A Python script was used to quantify the total number of alternations and to calculate the percentage of spontaneous alternation using the formula: (total number of alternations/number of arms entered) × 100% (Prieur & Jadavji 2019).

### Immunohistochemistry

Mice were anesthetized using isoflurane and cardiac puncture was performed during which mice were perfused with 20 ml of PBS. To ensure euthanasia, cervical dislocation was then performed. Mouse brain samples were hemisected on the sagittal midplane. The left hemispheres were fixed in 10% formalin, transferred to 70% ethanol and kept at 4 °C until paraffin embedding and tissue sectioning. The 5-μm sections were stained anti-CD68 antibody at a dilution of 1:300 (Cat# ab283654; Abcam, Waltham, MA) at the Research Histology Core Laboratory at the University of Texas MD Anderson Cancer Center (Houston, TX).

Brightfield microscopy was performed under a 10 × objective (Nikon Plan APO λD, 10x/0.45), with the BC43 Andor Benchtop Confocal Microscope (Oxford Instruments, High Wycombe, UK). A macro created in ImageJ (version 1.54p) was used to convert the z-stack into a single 2D image using the Z-projector plugin with the minimum intensity value and to create a mask of only positive staining by thresholding. This macro was applied to all images such that the thresholding did not change between groups. A region of interest (ROI) was then created in ImageJ and applied to all images such that cornu ammonis 1 (CA1) of the hippocampus was in the ROI. Images where CA1 could not be identified were not included. Analysis of particle counts was then run in the ROI with the same parameters for all images (circularity 0.10—1, size 5—200). For figure generation images were taken in 10 × using a Nikon Plan Flour 10x/0.30 in the Nikon ECLIPSE Ti, with the Nikon DS-Fi2 using NIS elements (version 6.10.01).

### Statistical analysis

Weights were analyzed with a two-way mixed-effects analysis followed by Tukey’s multiple comparisons test. The results of the behavioral tests were analyzed with a two-way ANOVA followed by Tukey’s multiple comparisons test. CD68 staining data were analyzed for outliers using the ROUT method with an alpha of 0.05, followed by two-way ANOVA and Tukey’s multiple comparisons test. These analyses were completed using GraphPad Prism (ver. 10.6.1; Boston, MA). Data are presented as mean ± SEM. P < 0.05 was considered to be statistically significant.

## Results and discussion

### Synthesis of C_60_-ser

**Fig. 1:**
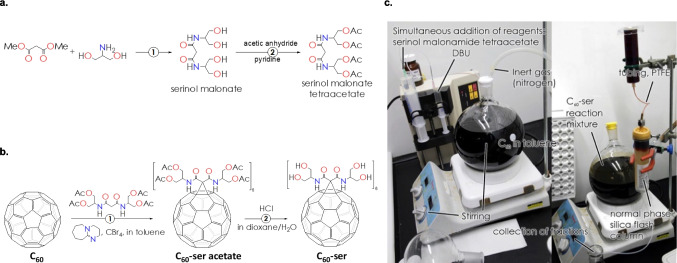
Synthesis of C_60_-ser. (**a**) A two-step synthesis to serinol malonamide tetraacetate. (**b**) Synthetic route to C_60_-ser. (**c**) Experimental setup of C_60_-ser synthesis. *Left*, simultaneous addition of reagents into the flask with C_60_ solution in toluene. *Right*, the absorption of reaction products on liquid chromatography column

C_60_-ser was synthesized through cyclopropanation of [60]fullerene in a two-step process based on our previous published method with significant improvements (Lee et al. 2009). In the first step, serinol malonamide tetraacetate was prepared by reaction between serinol and dimethyl malonate to form serinol malonate, which then undergo acetylation to form serinol malonamide tetraacetate (Fig. 1a). In the second step, C_60_ was allowed to react with the serinol malonamide tetraacetate in the presence of a DBU base (1,8-diazabicyclo[5.4.0]undec-7-ene)(Fig. 1b-c). The resulting C_60_-ser acetate product, a hexakis-adduct, was isolated by using liquid chromatography and subjected to *O*-deacetylation. Solid C_60_-ser was then dissolved in distilled water, neutralized to pH 6.5, purified by dialyses in deionized water, and stored in a solid form at 4 °C after vacuum drying. Determination of C_60_-ser purity was performed using MALDI ToF mass spectrometry and HPLC. Chromatographic peaks were characterized by the retention time (*t*_*R*_), width at ½ of peak height (*w*_*½*_), and quantified with peak area (*A*). By comparing peak areas, the C_60_-ser sample purity was 97.3% for hexakis-adduct and 2.7% for pentakis-adduct.

### No overt signs of toxicity after C_60_-ser administration in vivo

**Fig. 2:**
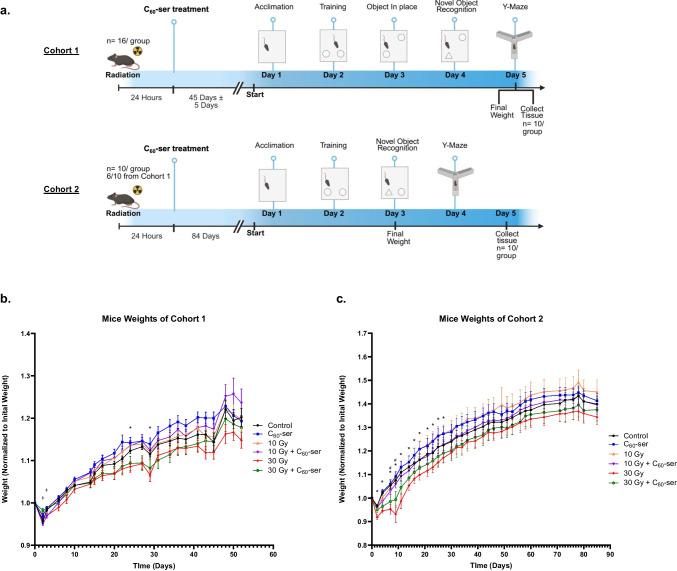
Normalized body weight throughout the experimental period. (**a**) Timeline and design of the experiment. Twenty-four hours after irradiation at 10 or 30 Gy, mice were given C_60_-ser (700 mg/kg) or 100 μl PBS orally. For cohort 1 (16 mice/treatment group), a battery of behavioral tests were performed 45 ± 5 days after C_60_-ser administration. Mice were divided into batches, with each batch containing an equal number of animals from each treatment group. Behavioral testing was conducted batch by batch, and all mice within a given batch completed the full testing schedule together. For cohort 2 (10 mice/treatment group), 6 out of 10 mice had previously been part of cohort 1 and had already undergone the behavioral tests. The remaining 4 mice had not been subjected to the tests prior to this cohort. Mice weights were measured 3 times a week. Brain tissues were harvested after completion of the test battery. (**b—c**) Body weights of mice shown in (**b**) cohort 1 and (**c**) cohort 2 were normalized to each mouse’s initial body weight prior to irradiation (mean ± SEM). A two-way mixed-effects analysis followed by Tukey's post hoc test was performed. (**b**) *, timepoint when 30 Gy was significantly lower than another group; ‡, timepoint when 10 Gy + C_60_-Ser was significantly lower than another group. (**c**) *, timepoint when 30 Gy was significantly lower than another group; #, timepoint when 30 Gy + C_60_-Ser was significantly lower than another group. p < 0.05 was considered statistically significant

We examined the effects of C_60_-ser on cognitive performance and neuroinflammation in mice following a single cranial radiation exposure. The overall experimental design is illustrated in Fig. 2a. Two cohorts of mice were included in the study, with cognitive assessments conducted either 45 ± 5 days or 84 days after C_60_-ser administration. All mice survived to the experimental endpoints with the exception of one animal that failed to recover from ketamine anesthesia. The remaining animals displayed normal feeding behavior and activity, showed no observable signs of distress or toxicity, and gained weight throughout the study regardless of treatment group.

In the first cohort, body weight was monitored until endpoint (45 or 52 days) following treatment (Fig. 2b). To avoid double counting, mice from cohort 1 who were also part of cohort 2 were not included. Two-way mixed-effects analysis revealed a significant effect of treatment on weight gain (body weight at a particular time point normalized to initial weight) (F = 3.408, p = 0.0223), as well as a significant interaction between treatment and time (F = 2.865, p = 0.0321), indicating that weight gain differed among treatment groups and that these differences varied across the observation period. A post hoc Tukey’s comparison analysis demonstrated that mice receiving 30 Gy of radiation exhibited significantly reduced proportional weight gain compared with other groups at multiple time points. Intriguingly, mice receiving 30 Gy followed by C_60_-ser treatment did not exhibit this reduction, suggesting a protective effect of C_60_-ser on overall physiological status; however, this difference did not reach statistical significance. A similar non-significant increase in proportional weight gain was observed with C_60_-ser treatment in the 10 Gy group compared with 10 Gy-only controls.

In the second cohort, in which body weight was monitored for 84 days, mixed-effects analysis did not identify treatment group as a significant factor influencing proportional weight gain (F = 2.076, p = 0.1218) (Fig. 2c). The 30 Gy radiation group consistently showed the lowest weight gain, whereas mice receiving 30 Gy and C_60_-ser demonstrated relatively greater weight gain (not statistically significant). At the final time point in both cohorts, normalized weight gain did not differ significantly among groups (Fig. 2b and c). Overall, 30 Gy radiation was associated with reduced weight gain, while C_60_-ser treatment showed a consistent but non-significant trend to mitigate these effects.

### C_60_-ser mitigates radiation-induced behavioral deficits

**Fig. 3:**
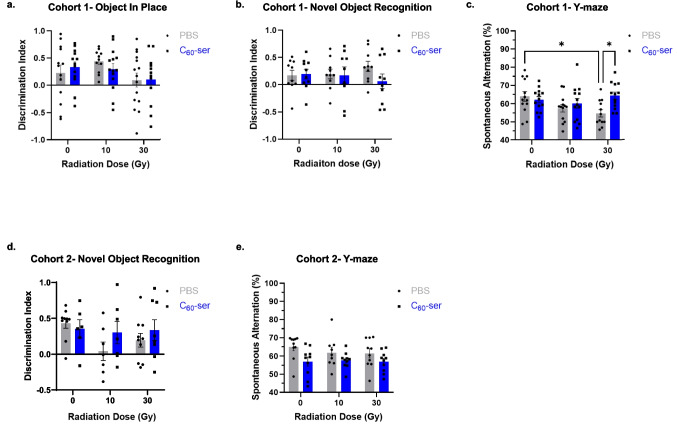
Analysis of mouse performance across the behavioral test battery. Mice were subjected to the indicated behavioral tests. Test performance of mice in (**a—c**) cohort 1 and (**d—e**) cohort 2 is shown. Mice with less than 9 s of exploration time in the object in place or novel object recognition tests were excluded from the analysis. Data are presented as mean ± SEM. Statistical analysis was performed using two-way ANOVA with Tukey’s post hoc test. *, p < 0.05. Pairwise comparisons without an asterisk indicate no statistically significant difference

Behavioral performance was evaluated using behavioral test batteries consisting of OIP, NOR, and Y-maze tests. Analysis of the mice exploration time on the training day indicated that the mice received similar training (Supplementary Fig. 1). The OIP task, which assesses memory for both object identity and object placement, is dependent on the neural circuit between medial prefrontal cortex, perirhinal cortex, and the hippocampus (Barker et al. 2007; Barker & Warburton 2009). A higher discrimination index is interpreted as an indicator of better associative recognition memory. The task performed on day 45 yielded mean discrimination indices of 0.2273, 0.4408, and 0.0940 for the control, 10 Gy, and 30 Gy groups, respectively (Fig. 3a). The corresponding C_60_-ser-treated groups showed mean discrimination indices of 0.3338, 0.2937, and 0.1071. A two-way ANOVA showed no significance effect due to either radiation (F = 2.651, p = 0.0775) or C_60_-ser administration (F = 0.008939, p = 0.9249). Post hoc test showed no significant differences among groups.

The NOR test assesses recognition memory which primarily depends on the perirhinal cortex (Barker et al. 2007; Ennaceur 2010), whereas the involvement of hippocampus is more controversial (Cohen & Stackman 2015). The value of discrimination index reflects the difference between the time spent on the familiar object and the novel object. Higher positive value signifies a preference for the novel object and better recognition memory. A two-way ANOVA showed no significance effect due to either radiation (F = 0.015, p = 0.9853) or C_60_-ser administration (F = 1.038, p = 0.3130) (Fig. 3b). Post hoc test revealed that there was no significant difference between any groups.

The Y-maze spontaneous alternation behavior test evaluates spatial working memory, which is dependent on the hippocampus and the prefrontal cortex (Kraeuter et al. 2019; Prieur & Jadavji 2019). A higher spontaneous alternation is interpreted as an indicator of better spatial working memory. Notably, we observed a dose-dependent reduction in alternation percentage following radiation exposure, with mean values of 63.93%, 57.50%, and 54.69% for the 0 Gy, 10 Gy, and 30 Gy groups, respectively (Fig. 3c). In contrast, C_60_-ser-treated groups-maintained alternation percentages above 60%, with mean values of 62.19%, 60.21%, and 64.28% for the 0 Gy, 10 Gy, and 30 Gy groups, respectively. In this task, a two-way ANOVA revealed that the interaction between radiation and treatment was significant (F = 3.355, p = 0.0405). Post hoc Tukey’s multiple comparison test showed that performance was decreased in mice exposed to 30 Gy radiation compared to the control (p = 0.0487) and recovered significantly in mice which received C_60_-ser after exposure to 30 Gy radiation compared to those which only received PBS (p = 0.0364). Treatment with C_60_-ser restored the behavioral performance of irradiated mice to a level that was statistically the same as the control mice (p > 0.9999).

The stronger protection effect of C_60_-ser seen in Y-maze than in OIP and NOR may implicate a higher degree of hippocampal involvement as the Y-maze evaluates spatial working memory which is sensitive to hippocampal damage (Kraeuter et al. 2019; Prieur & Jadavji 2019), whereas the other two tasks engage additional brain regions, including the prefrontal and perirhinal cortex (Alaghband et al. 2020). We also found that a 10 Gy dose of radiation had no detectable effect, whereas 30 Gy produced clear deficits. Although studies have reported behavioral impairments at 10 or lower Gy (Bekal et al. 2021; Raber et al. 2004; Son et al. 2015), there is considerable variability across the literature, making it difficult to identify a definitive benchmark for comparison. These discrepancies likely reflect differences in experimental design, including mouse strain, radiation parameters (dose rate and frequency of administration), and the specific behavioral tests, their setup, and the timing of assessment (Nigri et al. 2022).

A second cohort was assessed to determine whether the neuroprotective effects of C_60_-ser persisted at a later time point (84 days) following radiation (Fig. 3d-e). In the NOR test, groups exposed to radiation showed lower discrimination index values, although this effect was not dose dependent, with mean discrimination indices of 0.0399 for 10 Gy and 0.1930 for 30 Gy (Fig. 3d). Mice receiving C_60_-ser in combination with radiation displayed higher discrimination index values compared to their irradiated counterparts, with means of 0.3003 for 10 Gy + C_60_-ser and 0.3335 for 30 Gy + C_60_-ser. These values were closer to those observed in unirradiated mice (0.4297 for control and 0.3519 for C_60_-ser only). Despite these trends, there was no significance difference between radiation-only groups and their corresponding C_60_-ser-treated groups.

In the Y-maze task, the dose-dependent radiation-induced reduction in alternation observed at 45 days was no longer present at 84 days post-exposure (Fig. 3e). Mean alternation percentages were comparable across radiation groups, measuring 64.97% in controls, 61.89% in the 10 Gy group, and 61.46% in the 30 Gy group. C_60_-ser-treated mice exhibited alternation rates of 56.93%, 57.61%, and 56.88% in the 0 Gy, 10 Gy, and 30 Gy groups, respectively. No significant differences were detected between C_60_-ser-treated and untreated counterparts at this later time point.

Overall, behavioral testing demonstrated that radiation exposure impaired spatial working memory that was ameliorated by C_60_-ser treatment, whereas statistically significant behavioral differences were not detectable at a later time point. It remains unclear why the cognitive deficit observed at earlier time points was no longer detectable on day 84. The cohort assessed on day 84 included animals that had previously undergone behavioral testing at day 45. To address the possibility that prior testing influenced later performance, the performance of mice that underwent two rounds of the test battery was compared to that of mice that underwent only one round (Supplementary Fig. 2); however, the results were comparable, suggesting that repeated testing did not account for the disappearance of the deficit at day 84. Although it has been suggested that repeated exposure to the same behavioral task can introduce carryover effects, such effects are generally mitigated by sufficiently long inter-test intervals (Cnops et al. 2022). Intervals of at least four weeks are commonly recommended, and studies employing intervals ranging from one to five months have successfully tracked persistent cognitive deficits in disease models without compromising alternation performance (Cnops et al. 2022). Given that our experimental design incorporated an inter-test interval exceeding four weeks, it is unlikely that carryover effects fully explain the absence of detectable deficits on day 84. Alternatively, behavioral function may partially recover over time, leading to reduced or undetectable deficits at later time points. Although it remains unclear whether this recovery is permanent and the mechanisms by which it occurs, functional plasticity and cognitive recovery has been observed in a subset of human cancer patients 4—6 months following radiation treatment (Chen et al. 2017; Cherng et al. 2026).

### C_60_-ser treatment rescues radiation-induced microglia activation

**Fig. 4:**
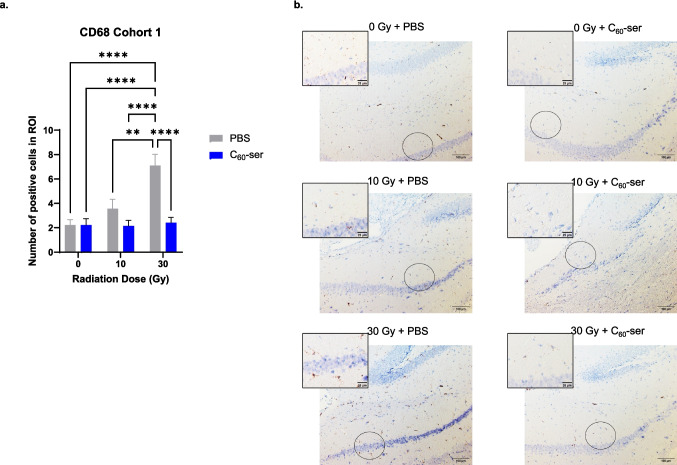
CD68 immunohistochemical staining in brain tissues of mice in cohort 1 (endpoint at day 45). Brain tissues were stained for CD68. (**a**) The numbers of CD68-positive microglial cells are presented as the means ± SEM (n = 7—10). (**b**) Images of the hippocampus were acquired at 10 × magnification. Scale bar, 100 μm. The circles indicate the cornu ammonis 1 (CA1) regions magnified in the inset panels. The zoomed inset images define the regions of interest (ROI) used for the quantification of CD68-positive microglial cells. Scale bar, 25 μm. Statistical analysis was performed using two-way ANOVA with Tukey’s post hoc test. **, p < 0.01; ****, p < 0.001. Pairwise comparisons without an asterisk indicate no statistically significant difference

**Fig. 5:**
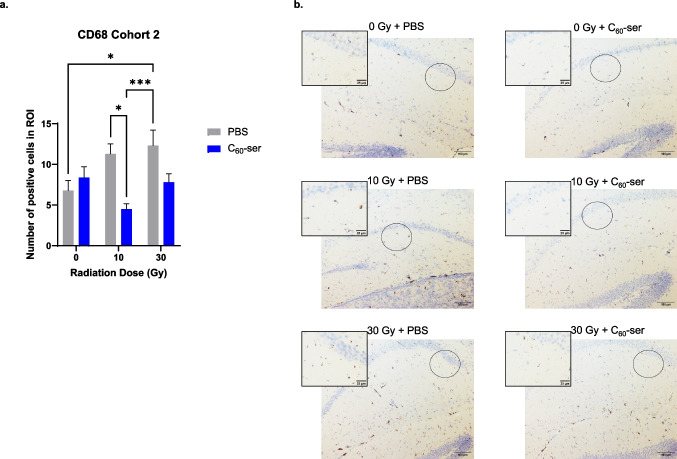
CD68 immunohistochemical staining in brain tissues of mice in cohort 2 (endpoint at day 84). Brain tissues were stained for CD68. (**a**) The numbers of CD68-positive microglial cells are presented as the means ± SEM (n = 7—10). (**b**) Images of the hippocampus were acquired at 10 × magnification. Scale bar, 100 μm. The circles indicate the cornu ammonis 1 (CA1) regions magnified in the inset panels. The zoomed inset images define the regions of interest (ROI) used for the quantification of CD68-positive microglial cells. Scale bar, 25 μm. Statistical analysis was performed using two-way ANOVA with Tukey’s post hoc test. *, *p* < 0.05; ***, *p* < 0.005. Pairwise comparisons without an asterisk indicate no statistically significant difference

Radiation triggers persistent neuroinflammation which leads to cognitive dysfunction (Lumniczky et al. 2017; Makale et al. 2017; Son et al. 2015). Therefore, the effect of C_60_-ser on microglial reactivity was examined in brain tissues collected from mice in both cohorts using the marker CD68 (Hendrickx et al. 2017; Lier et al. 2021). Since the behavior tasks rely primarily on memory, we focused on the cornu ammonis 1 (CA1) within the hippocampus as the region of interest. The CA1 region plays a central role in integrating hippocampal circuitry for memory consolidation and retrieval; inflammation in this area disrupts memory by activating microglia and altering neuronal synaptic function (Bartsch et al. 2011; Ziehn et al. 2010). The CA1 region has also been established to be most sensitive hippocampal region to damaging insults (Davidson & Stevenson 2024; Nairuz et al. 2024). Immunohistochemistry staining with anti-CD68 antibody and subsequent quantification revealed an elevated number of CD68-positive (CD68 +) cells upon radiation in a dose dependent manner, while tissues from mice receiving radiation and C_60_-ser had lower numbers of positive cells in both cohorts (Figs. 4 and 5). Although there were no significant effects of radiation on the cognitive behaviors at day 84 as shown in Fig. 3, the staining data indicated a sustained neuroinflammation that persisted for 84 days. Similar discordance between neural tissue biology and behavioral tests in mouse model has been reported, in which radiation caused loss of neurogenesis but did not result in impaired behavior and cognition (Kuil et al. 2024).

In cohort 1, a two-way ANOVA showed that radiation alone (F = 8.733, p = 0.0006), C_60_-ser treatment alone (F = 14.610, p = 0.0004), and the interaction between C_60_-ser treatment and radiation (F = 7.333, p = 0.0017) had significant impacts on the number of CD68 + cells. The number of CD68 + cells was significantly greater in the 30 Gy + PBS group compared to all other groups (p < 0.005) (Fig. 4). Remarkably, the 30 Gy + C_60_-ser group had significantly less CD68 + cells compared to 30 Gy + PBS group, resulting in no significant difference between 30 Gy + C_60_-ser and un-irradiated control (p > 0.9999). In cohort 2, a two-way ANOVA did not reach significance for radiation (F = 2.203, p = 0.1215) but showed that C_60_-ser administration significantly (F = 8.955, p = 0.0044) affected CD68 immunoreactivity. It also showed that the interaction between C_60_-ser and radiation was significant (F = 5.143, p = 0.0095). C_60_-ser treatment reduced CD68 + cell populations following irradiation; the reduction observed between the 10 Gy + PBS and 10 Gy + C_60_-ser groups was statistically significant (p = 0.0121) (Fig. 5). The difference between 30 Gy + PBS and 30 Gy + C_60_-ser did not reach significance (p = 0.1272). Together, these findings suggest that C_60_-ser treatment attenuates radiation-induced activation of microglia, thereby reducing neuroinflammation.

The data on improved behavioral performance associated with the suppression of microglial activation imposed by C_60_-ser are encouraging. Studies have shown that eliminating microglia after brain injury or in neurodegenerative disease model significantly improves cognitive recovery by reversing chronic neuroinflammation (Rice et al. 2015; Z. Zhang et al. 2025). Our data provide support for the strategy and demonstrate that C_60_-ser is a viable means of achieving this.

## Conclusion and outlook

Both the behavioral outcomes and molecular marker analyses consistently demonstrate a mitigative effect of C_60_-ser on radiation-induced cognitive dysfunction and neuroinflammation. This mitigative mechanism is likely mediated through suppression of microglial activation, thereby reducing neuroinflammation. Together, these findings establish a foundation for further investigation of the in vivo mitigative effects and the mechanistic actions of C_60_-ser in the context of radiation-induced neurotoxicity. Radiomitigative drugs have applications beyond traditional clinical settings. With growing interest in human space exploration, especially long-duration missions into deep space, radiation exposure has become a major concern (Fogtman et al. 2023). Biological countermeasures, including radiomitigative agents, can help reduce the health risks from cosmic radiation and enhance human safety during space missions.

Several considerations may strengthen future experimental designs. First, our experimental dose of 700 mg/kg did not show overt toxicity, but may be more than the required dose to see benefits, thus future studies could be used to determine more optimal dosing. Second, inclusion of female mice would improve sex balance and address potential sex-specific effects, as previous studies have suggested that female rodents may outperform males in memory tasks, particularly object recognition and spatial location, potentially due to the influence of ovarian hormones (Cost et al. 2012). Third, given that both our data and prior studies implicate the hippocampus in mediating radiation-induced neurological changes, additional hippocampal-dependent behavioral tasks, such as the Morris water maze, could provide complementary insights (Morris 1984). Fourth, additional molecular investigations are warranted to identify the specific inflammatory cell populations within the neural microenvironment that contribute to the observed protective effects and how they respond to C_60_-ser at different ages. Fifth, experiments with primary culture of neural stem cells from the dentate gyrus of the hippocampus may allow for direct examination of the effects of C_60_-ser on hippocampal neurogenesis (Ahmed et al. 2021; Babu et al. 2007). Finally, given that radiation treatment is evolving with the addition of new modalities such as proton beams, and neutron capture therapy, studying C_60_-ser in conjunction with those would expand the therapeutic role of radiomitigative drugs (Seneviratne et al. 2023; Yan et al. 2023).

## Supplementary Information

Below is the link to the electronic supplementary material.

Supplementary file1 (PPTX 84 KB)

## Data Availability

Data supporting the findings of this study are available within the paper and its Supplementary Information. Data are available from the authors upon reasonable request and with permission from the University of Texas Health Science Center.
