# Supplementary material for: Water-soluble fullerene derivatives mitigate cranial radiation-induced neuroinflammation and cognitive dysfunction
Source: Biomed Microdevices. 2026 May 11;28(2):36. doi: 10.1007/s10544-026-00818-w (PMC13161026; doi:10.1007/s10544-026-00818-w)
Supplement: Supplementary file 1 — Supplementary file1 (PPTX 84 KB) [file 10544_2026_818_MOESM1_ESM.pptx]

## Slide 1
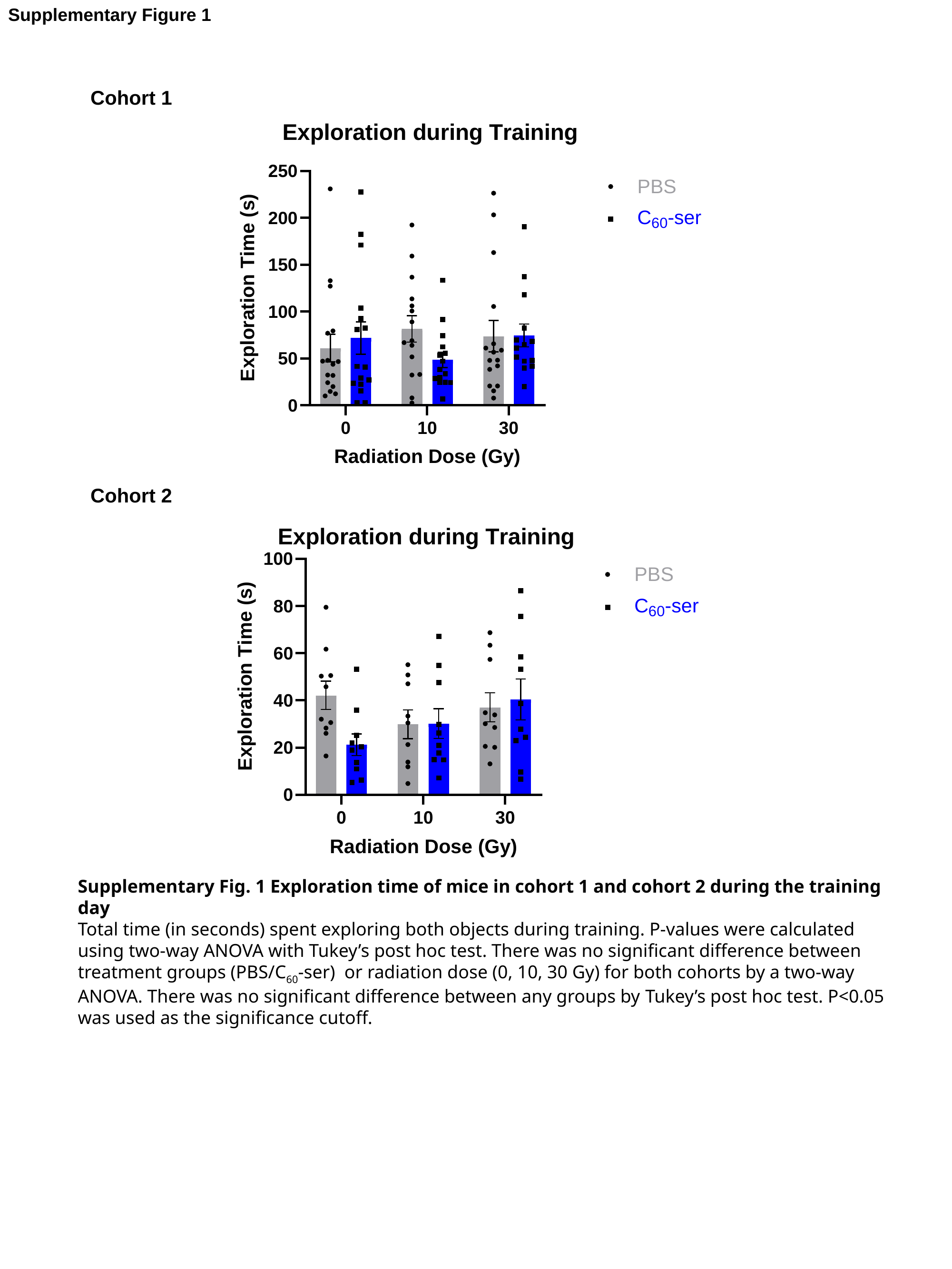

Supplementary Figure 1
Cohort 1
Cohort 2
Supplementary Fig. 1 Exploration time of mice in cohort 1 and cohort 2 during the training day
Total time (in seconds) spent exploring both objects during training. P-values were calculated using two-way ANOVA with Tukey’s post hoc test. There was no significant difference between treatment groups (PBS/C60-ser)  or radiation dose (0, 10, 30 Gy) for both cohorts by a two-way ANOVA. There was no significant difference between any groups by Tukey’s post hoc test. P<0.05 was used as the significance cutoff.

## Slide 2
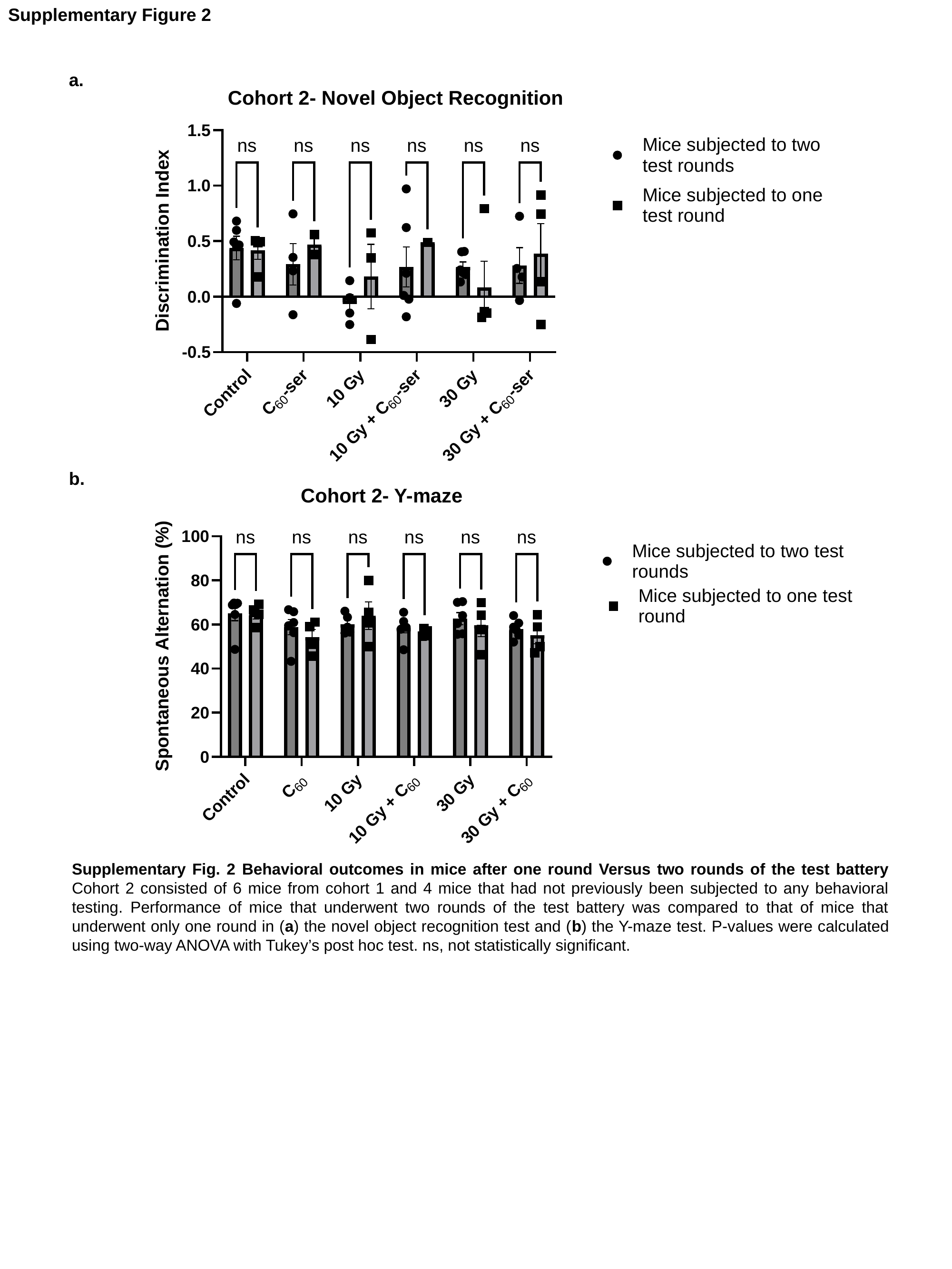

Supplementary Figure 2
a.
Cohort 2- Novel Object Recognition
b.
Cohort 2- Y-maze
Supplementary Fig. 2 Behavioral outcomes in mice after one round Versus two rounds of the test battery Cohort 2 consisted of 6 mice from cohort 1 and 4 mice that had not previously been subjected to any behavioral testing. Performance of mice that underwent two rounds of the test battery was compared to that of mice that underwent only one round in (a) the novel object recognition test and (b) the Y-maze test. P-values were calculated using two-way ANOVA with Tukey’s post hoc test. ns, not statistically significant.
